# Johan Mackenbach, awarded an honorary doctorate for his work on health inequalities, in a discussion of burning issues in tackling health inequalities

**DOI:** 10.1186/s12939-015-0242-3

**Published:** 2015-10-17

**Authors:** Vincent Lorant, William D’Hoore

**Affiliations:** IRSS, Institute of Health and Society, Université catholique de Louvain clos Chapelle-aux-Champs, 30 bte B1.30.15, 1200 Brussels, Belgium; IRSS, Institute of Health and Society, Université catholique de Louvain clos Chapelle-aux-Champs, 30 bte B1.30.16, 1200 Brussels, Belgium

**Keywords:** Health inequalities, Health policies, Stakeholders

## Abstract

On 20 March 2015, Professor Johan Mackenbach of the Erasmus University Medical Centre was awarded a doctorate *honoris causa* by the Catholic University (Université Catholique) of Louvain, Belgium, for his outstanding contribution to the analysis of health inequalities in Europe and to the development of policies intended to address them. In this context, a debate took place between Professor Mackenbach, Professor Maniquet, a well-being economist, and a representative of the Federal Health Ministry (Mr. Brieuc Vandamme). They were asked to debate on three topics. (1) socio-economic inequalities in health are not smaller in countries with universal welfare policies; (2) Policies needs to target either absolute inequalities or relative inequalities; (3) The focus of policies should either address the social determinants of health or concentrate on access to health care. The results of the debate by the three speakers highlighted the fact that welfare systems have not been able to tackle diseases of affluence. Targets for health policies should be set according to opportunity cost: health care is increasingly costly and a focus on health inequalities above all other inequalities runs the risk of taking a dogmatic approach to well-being. Health is only one dimension of well-being and policies to address inequality need to balance preferences between several dimensions of well-being. Finally, policymakers may not have that much choice when it comes to reducing inequality: all effective policies should be implemented. For example, Belgium and other European countries should not leave aside health protection policies that are evidence-based, in particular taxes on tobacco and alcohol. In his final contribution, Professor Mackenbach reminded the audience that politics is medicine on a larger scale and stated that policymakers should make more use of research into public health.

## Introduction

JM is Professor of Public Health at the Erasmus University Medical Centre, Rotterdam, in the Netherlands. JM has contributed significantly to the description, monitoring, and analysis of health inequalities. He and his colleagues have drawn attention to the higher risk of poor health among lower socio-economic groups than among higher socio-economic groups in almost all European countries, not only for mortality and morbidity but also for subjective appraisal of health [1–3]. By analysing inequalities across different causes of death, he showed that these inequalities may have different causes across countries [1, 3, 4]. Tackling health inequalities may thus require different approaches in different countries. Health inequality is not a disappearing issue: JM has shown that the gap between socio-economic groups has not decreased or has even increased over time [5, 6]. Disparities in life expectancy between European countries have increased and are now as large as those found just after the Second World War [7]. Health inequality, moreover, is a multidimensional problem: occupation and education were for a long time the main social determinants analysed. More recently, however, JM has acknowledged the role of ethnicity and migration as other important determinants, particularly in a globalised world [8]. Another important contribution was to move away from description and to pay attention to both risky behaviour [9, 10] and healthy behaviour [11]. As these behaviours are affected by public policy, he scrutinised the performance of public health policies in Europe and found that, for a given income level, some countries, including a rich country like Belgium, could do much better [12, 13]. He thus helped to put policies and political factors [14, 15] back on the research agenda, particularly in the last decade when economic crises were putting our health care system at risk [16].

As a consequence of this impressive contribution to the field, the Catholic University of Louvain (UCL) awarded, Professor Johan Mackenbach (JM), on the 20^th^ of March 2015, an honorary doctorate for his outstanding contribution to research and policymaking in the field of health inequality. The UCL also praised his efforts to carry out interdisciplinary research combining public health and social science research to come up with a nuanced, complex, and non-dogmatic perspective on the causes of and solutions to health inequalities here and elsewhere. He has contributed to the health inequality agenda in a way that has made health inequalities a mainstream, interdisciplinary topic of research, reaching out beyond the traditional borders of public health research. More recent publications by Professor Mackenbach have shown how inequality is also becoming an issue in political science, not only because public health researchers have sought to influence policy but also because health policies may be part of the problem: for example, research about tobacco and alcohol control policies has shown that some government have been slow or reluctant to enact legislations protecting health and supporting healthy choices [13, 17].

The landmark WHO report on the Social Determinants of health “Closing the Gap in a Generation” has contributed to move on towards a clear perspective on how best to tackle health inequalities and identifyied strategies to reduce health inequalities, such as improving the daily life conditions, improving income redistribution or monitoring the problem [18]. JM has also made a major contribution to the design and assessment of such strategies [19, 20]. But whether resources are made available and whether policymakers are willing to follow suit remains to be seen. To put JM’s contribution into a broader societal perspective, a debate was organised with two other speakers, who provided complementary perspectives from the fields of economics and policymaking. This commentary summarises this debate on how best to tackle health inequalities. Professor Mackenbach was asked to debate with two others, Professor François Maniquet (FM), an economist in the fields of well-being and social responsibility who has contributed to the discussion of fairness and welfare [21], and Brieuc Vandamme (BVD), deputy director of the minister’s staff at the Federal Health and Social Affairs Ministry, Belgium. The debate took place on 20 March and was chaired by Vincent Lorant (VL).

## Three burning issues in tackling health inequalities

The participants were asked to address three general subject areas: the welfare paradox, targets to reduce health inequalities, and the question of which policies should be given the priority.

### Why health inequalities have not disappeared in welfare states ?

Welfare regimes, in many countries, have contributed to the reduction of income inequalities between social strata, but have not contributed so much to the reduction of health inequalities [22]. How can we explain and resolve this paradox? JM emphasised two key elements that help to explain the persistence of inequality. He first noted that even countries with well-developed social welfare systems have not eliminated inequalities in material well-being, such as economic resources or capital (i.e. income, housing). The second key element is that social mobility has become more selective with regard to health and health-related factors. Moreover, welfare systems have been designed to tackle diseases related to poverty, not the diseases of affluence that have become common since the epidemiological transition, such as cardiovascular disease or cancer.

The federal government representative, BVD, mentioned that most disease risk factors were beyond the reach of health care, which is a federal competence in Belgium, whereas health prevention and promotion were competences of the regional authorities. This statement clearly calls for a better coordinated action across the different public agencies involved in health and health care issues, something which is an issue in Belgium or in other federal countries, counting with multiple layers of power.

In addition, according to FM, welfare systems have been designed to redistribute income and the relevant question is whether health inequalities before tax are higher than health inequalities after tax. This last point refers clearly to the question of whether lower income inequality (partly because of a more redistributive tax system) is associated with less health inequality, an issue which is still the subject of much debate in public health circles [23–25]. Professor Mackenbach insisted that government is not only responsible for equal access to health care but also be concerned with the social distribution of other factors related to health.

### Targeting health inequalities: absolute health inequalities or relative inequalities?

The trend in health inequalities is not encouraging: some Western countries with strong and long-standing welfare systems have either reported a widening gap in life expectancy across educational groups [26, 27] or no narrowing across geographical regions [28]. In general, absolute inequalities, defined as differences in mortality rates or in disease prevalence between socio-economic groups, have decreased over time, whereas relative inequalities (the ratio of the mortality of the lower socio-economic group to the mortality of the higher socio-economic group) have been maintained or have even increased over the last decade. Which would be the right target for policies: to reduce absolute inequalities or relative inequalities?

According to the FM, this question was impossible to answer: it all depends on preferences. Indeed, there is no reason why preferences for health should take precedence over other kinds of well-being. Are we willing to devote vast amounts of money to one dimension of well-being (health), rather than to others, such as income or education? Claiming that tackling health inequalities is “the right thing to do” is a dogmatic and paternalistic approach that assumes that someone knows better. One possible way to investigate such choices would be to assess whether there is a trade-off between health and other socially valued kinds of well-being. The question of cost was also emphasised by BV, particularly in the context of the rising cost of health care. A more pragmatic choice would be to target absolute inequalities, which would benefit all population groups. JM reminded the audience that when the WHO-Europe Region updated its “Health for All” targets, the target relating to equity was rephrased as follows: “By the year 2020…the gap in life expectancy between socioeconomic groups should be reduced by at least 25 %” [29], leaving unanswered the question of whether it meant absolute inequality or relative inequality. If the WHO meant an absolute inequality gap, then an improvement of the life-expectancy in the general population is consistent with such target. On the opposite, attaining a decrease of 25 % in the relative gap of life-expectancy, would require flattening the curve between life-expectancy and, say, socio-economic status: this implies different efforts and re-allocation of resources across socio-economic groups , including a more focused approach on the structural determinants of health, which is the next contentious topic.

### What should be the policy priorities to reduce health inequalities: health care, health behaviour, or structural determinants?

For the first time, the Belgian Federal Government declaration of October 2014 addressed the question of health inequalities in the following terms: “the Government will take initiatives necessary to tackle this phenomenon [life expectancy inequalities] and will undertake concrete actions to prevent the increase of socio-economic differences in health and to work for a substantial reduction of health care socio-economic inequalities in different domains.” [30] The scientific literature suggests that health care is just one modest factor contributing to health. Should-we aim to reduce inequalities in health care, or in health behaviours (for example by heavier taxation of tobacco products), or in the structural determinants of health (education, income, employment, etc.)? BV emphasised two priorities from the point of view of the government: improving access to health care and reducing financial barriers to health care. He also suggested that providing patients with good information would help them make appropriate choices, for example between services providing different quality of care. He did not mention the structural determinants of health. JM suggested that we may not have the luxury of choosing among options and that we should use all available and evidenced-based strategies to tackle inequalities in and outside the health care sector: we need all these measures if we want to make a difference in health inequalities. In the specific case of Belgium, JM mentioned the need to improve cancer screening and counselling for tobacco cessation. According to FM, reducing health inequalities benefits the well-being of all citizens, but well-being has several dimensions and the choices to be made in health care, education, and other areas of public spending need to be based on information about their contribution to these different dimensions of well-being. The choices should be made according to citizen preferences and not according to an overarching principle that places heath above everything else. BV was supportive of that perspective, saying it was a moral duty of policymakers to be effective across these different kinds of expenditure.

## Conclusion

This debate yielded three key results. First, health inequalities look less “unfair” from outside the field of public health than from inside, because health is just one dimension of well-being. Public health advocates or scholars tend to consider health as a prominent “good” which cannot be traded against other social goods. Yet, when having to allocate resources, policymakers and citizens may not value health disregarding other important socially valued outcomes such as education and income.

Secondly, it is likely that absolute inequalities are what policymakers have in mind when they discuss inequality. As a consequence, generic approaches are thus more likely to be supported by policymakers. That kind of approach, however, fails to address the question as to which policies are desirable and to consider the need for radical solutions, which may involve inter-sectoral policies. Is it time for structural changes? There seems, at present, to be a gulf between one possible solution (improving access to health care, i.e. reducing inequalities in terms of disease) and the range of questions that health inequalities raise about our societies.

Finally, BV, the policymaker, and FM, the welfare economist, seemed to share the opinion that tackling health inequalities was also a matter of choice. Health, as the paramount “good”, should not be taken for granted by public health scholars or advocacy groups. What are citizens and policymakers willing to do to lower health inequalities? How do citizens trade off different dimensions of well-being, including, but not exclusively, health? Do they continue to accept that the priority is not health (but may, for example, be education and employment) and do they accept health inequalities as side effects of other policies intended to reduce inequality?

The role of public health research is to help clarify these questions and also to help in getting evidence-based research carried out. For many countries, including Belgium, there remains room for improvement, particularly in the domain of health protection. To conclude with JM’s words, “Politics is medicine on a larger scale.” Public Health researchers should therefore get more involved in public policy.
